# Clinical and Biological Determinants of Longitudinal Cognitive Function in Patients With *GBA1* Variants and Subthalamic Deep Brain Stimulation

**DOI:** 10.1002/ana.78139

**Published:** 2026-01-16

**Authors:** Moritz A. Loeffler, Philipp Klocke, Isabel Wurster, Stefanie Lerche, Idil Cebi, Thomas Gasser, Alireza Gharabaghi, Kathrin Brockmann, Daniel Weiss

**Affiliations:** ^1^ Centre for Neurology, Department of Neurodegenerative Diseases University of Tübingen Tübingen Germany; ^2^ Hertie‐Institute for Clinical Brain Research University of Tübingen Tübingen Germany; ^3^ Institute for Neuromodulation and Neurotechnology University of Tübingen Tübingen Germany

## Abstract

**Objective:**

Whether cognitive decline in patients with Parkinson's disease (PD) carrying *GBA1* variants is accelerated after subthalamic deep brain stimulation (STN‐DBS) remains controversial. Clarifying long‐term cognitive outcomes is essential for informed decision making.

**Methods:**

We assembled matched cohorts of patients carrying *GBA1* variants with STN‐DBS (PD_GBA1+DBS+_, n = 28) and without (PD_G BA1+DBS−_, n = 28). Additional cohorts included non‐carriers with STN‐DBS (PD_GBA1−DBS+_, n = 40) and without (PD_GBA1–DBS−_, n = 43). Clinical, genetic, and cerebrospinal fluid (CSF) biomarkers (A*β*1–42, h‐Tau, p181‐Tau, and neurofilament light chain) were analyzed. Cognition was assessed using the Montreal Cognitive Assessment (MoCA). Cognitive slopes were estimated using linear mixed models and the minimally detectable slope difference at 3‐year follow‐up was 1.33 MoCA points enabling sensitivity to clinically meaningful changes. Secondarily, conversion to dementia was analyzed with Kaplan–Meier‐analysis once the MoCA was < 21.

**Results:**

There was no significant difference in cognitive decline between PD_GBA1+DBS+_ and PD_GBA1+DBS−_ (−0.24 MoCA points/year; 95% confidence interval [CI] = –1.11 to 0.70) projecting to −0.72 MoCA points at 3‐year‐follow‐up (*p* = 0.583). Secondarily, the risk for conversion to dementia did not differ between PD_GBA1+DBS+_ and PD_GBA1+DBS−_ (HR = 0.55, 95% CI = 0.23–1.34, *p* = 0.119) or between PD_GBA1−DBS+_ and PD_GBA1−DBS−_ (HR = 1.22, 95% CI = 0.53–2.83, *p* = 0.897). Dementia risk was associated with *GBA1* status (HR = 3.04, 95% CI = 1.05–8.79, *p* = 0.041), baseline MoCA < 26 (HR = 3.05, 95% CI = 1.29–7.21, *p* = 0.011), and baseline age > 69 years (HR = 4.42, 95% CI = 1.79–10.89, *p* = 0.001). In *GBA1* carriers, a visuospatial/executive domain score < 4/5 predicted dementia (HR = 4.71, 95% CI = 1.25–17.86, *p* = 0.022).

**Interpretation:**

*GBA1* variant carriers meeting general STN‐DBS indication criteria did not show accelerated cognitive decline in the presence of STN‐DBS. In addition, exploratory predictors of dementia could support counseling of DBS candidates. ANN NEUROL 2026;99:976–988

Heterozygous variants in the *glucocerebrosidase 1* (*GBA1*) gene represent the most important genetic risk factor for Parkinson's Disease (PD) occurring in 5% to 15% of people with PD.^1^ Individuals with PD carrying *GBA1* variants (PD_GBA1_) typically experience disease onset approximately 5 years earlier and exhibit faster disease progression. This includes earlier cognitive decline and a higher prevalence of PD‐related dementia (PD dementia).^1^, ^2^, ^3^, ^4^ *GBA1* encodes the lysosomal enzyme glucocerebrosidase, which metabolizes glucosylceramides and glucosylsphingosines. Variants in *GBA1* result in lower glucocerebrosidase activity and cause a build‐up of glucosylceramides and glucosylsphingosines.^2^ Consequently, lysosomal and possibly mitochondrial function are impaired promoting alpha‐synuclein aggregation.^3^

Due to the relatively early disease onset and frequent occurrence of motor fluctuations, patients with PD_GBA1_ exhibit a clinical phenotype of motor symptoms qualifying for deep brain stimulation of the subthalamic nucleus (STN‐DBS)^5^, ^6^, ^7^ provided that PD dementia, considered a contraindication for STN‐DBS,^8^ has not manifested. Therefore, the proportion of *GBA1* variant carriers in STN‐DBS implanted PD cohorts is higher than in the non‐DBS PD population ranging up to 20%.^6^, ^9^ Although there is consensus that patients with PD_GBA1_ benefit from STN‐DBS regarding improvement of motor fluctuations, as do patients without *GBA1* variants,^6^, ^10^, ^11^ its effect on longitudinal cognitive outcomes remained controversial after one previous study suggested more severe cognitive decline if PD_GBA1_ were treated with STN‐DBS.^12^ In this analysis of 12 aggregated datasets, 58 patients with PD_GBA1+DBS+_ showed a mean annual decline of 1.71 points more than seen in 82 PD_GBA1+DBS−_ patients determined by the Mattis Dementia Rating Scale (MDRS). Notably, the follow‐up period and disease duration were longer in the PD_GBA1+DBS+_ cohort compared with the PD_GBA1+DBS−_ cohort (53 vs 36 months; 11 vs 7 years). In general PD populations, negative effects on specific cognitive subdomains, namely on verbal fluency and executive control, have been observed following STN‐DBS implantation regardless of the genetic status.^13^, ^14^, ^15^, ^16^, ^17^, ^18^, ^19^ However, long‐term observations did not associate STN‐DBS with an increased risk of PD dementia.^17^, ^20^ Notably, the long‐term observational data were based on genetically unstratified STN‐DBS cohorts and compared to general PD population data, often lacking control cohorts.

These unsolved issues and the controversy in *GBA1* variant carriers left the DBS field with uncertainty how to manage PD_GBA1_ ^21^ and a more holistic understanding has not been achieved yet. As such, clinical variables impacting cognitive decline have not been disentangled from the variant carrier status so far. In addition, biological determinants of cognitive decline in PD, such as *APOE4* allele, *MAPT* haplotype,^22^, ^23^ and concomitant Alzheimer's pathology reflected by CSF profiles^23^, ^24^ have not been taken into account when making informed decisions toward STN‐DBS in patients with or without *GBA1* variants.

Because PD_GBA1+_ patients without manifest PD dementia present commonly for STN‐DBS counseling, a deeper understanding of the clinical, genetic, and biological factors determining PD dementia risk is crucial for informed decision making. Therefore, this study aimed to analyze the longitudinal cognitive outcome in well‐characterized matched cohorts of PD_GBA1_ variant carriers and non‐variant carriers, both with/without STN‐DBS based on genetic, demographic, clinical, and cerebrospinal fluid (CSF) parameters.

## Methods

### Study Cohorts

Over the last 20 years, our center has followed ~4,000 patients with PD within the Tuebingen Parkinson Cohort (TUEPAC). Standardized genetic, clinical, and biomarker data are stored for research purposes in the database of the Neurobiobank of the Hertie Institute for Clinical Brain Research (https://www.hih-tuebingen.de/ueber-uns/core-facilities/biobank/for-researchers). Supplementary Figure S1 summarizes the formation of the study cohort. For the present analysis, 511 patients with PD_GBA1_ were identified. Of those patients, 53 were implanted with STN‐DBS (PD_GBA1+DBS+_) between 2004 and 2023. Cognitive eligibility criteria for referral to STN‐DBS were in accordance with the CAPSIT‐PD guidelines,^25^ excluding individuals with overt dementia. Nevertheless, we identified 2 individuals who had received STN‐DBS in the early days of DBS surgery at our center despite a Montreal Cognitive Assessment (MoCA) < 21, currently considered a cutoff for PD dementia^26^, ^27^ who were excluded from the analysis. Patients with mild cognitive impairment were clinically considered for STN‐DBS based on individual risk–benefit assessment,^25^ and therefore included in the analysis. As research evolved over time, we became more reluctant to indicate patients with mild cognitive impairment for STN‐DBS given their less beneficial quality‐of‐life outcomes after STN‐DBS.^28^ In addition, we excluded patients with non‐pathogenic *GBA1* variants (n = 12) and those lost to follow‐up within 12 months from DBS surgery (n = 11). The final PD_GBA1+DBS+_ cohort comprised 28 patients.

Of the remaining patients in the PD_GBA1_ group without STN‐DBS, we matched a disease‐genetic‐control cohort: for each patient with PD_GBA1+DBS+_, we identified a patient matched for (i) the same *GBA1* variant severity, (ii) disease duration (±2 years), age (±5 years), and baseline cognitive function measured by the MoCA (±2 points) resulting in a PD_GBA1+DBS−_ cohort (n = 28).

Additionally, we included cohorts of wildtype PD with and without STN‐DBS matched to the mean disease duration (±2 years), age (±5 years), and baseline MoCA (±2 points) of the PD_GBA1+DBS+_ cohort: PD_GBA1−DBS+_ (n = 39), and PD_GBA1−DBS−_ (n = 43).

As simultaneous mutations of *GBA1* and *LRRK2* show a more benign cognitive outcome,^29^, ^30^ all patients were screened for simultaneous *LRRK2* mutations which were not observed. In addition, 117 of 138 patients were screened for pathogenic variants in *PRKN*, *PINK1*, and *SNCA*, which have been implicated in modifying the rate of cognitive decline^31^; no such variants were identified.

### Clinical Assessments

Cognitive function was assessed using the MoCA.^32^ As the Mini‐Mental State Examination (MMSE) was used in our center before 2014, in 34 of 139 cases, MMSE scores were subsequently transformed to MoCA scores according to a standardized algorithm.^33^ Patients’ motor status was evaluated using the Movement Disorder Society‐Unified Parkinson's Disease Rating Scales (MDS‐UPDRS) III and IV.^34^ Depressive symptoms were assessed by the Beck Depression Inventory (BDI‐II).^35^ The levodopa equivalent daily dosage (LEDD) was calculated according to published algorithms.^36^, ^37^ The occurrence of freezing of gait (FoG), falls, and hallucinations were recorded as binary outcome parameters.

### Genetic Analysis

DNA was isolated from ethylenediaminetetraacetic acid blood by the salting‐out method and stored at 4°C. Genetic screening of all exons of the *GBA1* gene and for pathogenic variants in *LRRK2*, *PINK1*, and *PRKN* was performed by Sanger sequencing and for copy number variants in *PRKN* and *PINK1* as well as duplications and deletions in *SNCA* by MLPA. Primers and conditions are available upon request. Genetic variants in *APOE* (rs429358 determining the E4 carrier status), *MAPT* (rs1052587 tagging the H1/H2 haplotype) have been assessed using NeuroChip.^38^ Variants in the *GBA1* gene were stratified according to mutation severity and classified as risk, mild or severe^39^ (Supplementary Table S1).

### CSF Collection

A spinal tap was performed between 9.00 am and 1.00 pm. Samples were centrifuged within 60 minutes and frozen at −80°C within 90 minutes from collection. Samples with abnormal routine CSF diagnostics (erythrocytes > 1/μl, white blood cell count > 5 cells/μl, and immunoglobulin subtype IgG index > 0.7) were excluded. CSF was available in 12 of 28 patients with PD_GBA1+DBS+_, 13 of 28 patients with PD_GBA1+DBS−_, 12 of 40 patients with PD_GBA1−DBS+_, and 19 of 43 patients with PD_GBA1−DBS−_.

### *CSF Measurement of Amyloid‐*β*_1‐42_, Total‐Tau, Phospho181‐Tau, and Neurofilament Light Chain*

CSF levels of amyloid‐*β*_1‐42_ (A*β*_1‐42_), total‐Tau (h‐Tau), and phospho181‐Tau (p181‐Tau) were measured using enzyme‐linked immunosorbent assay (ELISA) kits from INNOTEST (Fujirebio GmbH, Hannover, Germany). Our validated in‐house cutoffs for clinical routine indicating pathological levels are as follows: A*β*_1‐42_ < 600 pg/ml and p181‐Tau > 60 pg/ml. CSF levels of neurofilament light chain (NfL) were measured using the Uman Diagnostics NF‐Light assay (UmanDiagnostics, Umea, Sweden). Intra‐assay coefficients of variation for A*β*_1‐42_, p181‐Tau, and NfL were below 15%, respectively.

### Ethics Approval and Informed Consent

The study was approved by the Ethics Committee of the University of Tuebingen (26/2007BO1, 404/2010BO1, 199/2011BO1, and 702/2013BO1). All patients gave written informed consent.

### Statistical Analysis

Data management was conducted using the REDcap platform.^40^ Statistical analyses were performed using IBM SPSS Statistics 27 (IBM, Armonk, NY, USA), GraphPad PRISM 10 (GraphPad Software Inc, San Diego, CA, USA), the lm4 package in R software^41^ and MATLAB R2023b (MathWorks, Natick, MA, USA). Baseline paired cohort comparison of continuous variables was done using Wilcoxon signed rank test and across cohorts using the Mann–Whitney *U* test or Kruskal‐Wallis test.

As primary analysis, cognitive change between cohorts was estimated using linear mixed‐effects models with random intercepts and slopes, fitted by maximum likelihood estimation. This approach incorporated all available longitudinal MoCA data and enabled independent assessment of baseline cognition (intercept) and cognitive decline over time (slope). Analyses were conducted both unadjusted and adjusted for the logit of calculated propensity scores [log(propensity score / (1 – propensity score))], accounting for baseline differences in age at onset, age at baseline, MoCA, MDS‐UPDRS III, and LEDD. To evaluate statistical sensitivity, a post hoc power analysis was performed assuming 80% power, *α* = 0.05, a sample size of 28 (smallest groups), an average of 3 visits per patient, and the dataset's residual variance (3.64) and intraclass correlation coefficient (0.526).^42^ Aiming for a conservative approach, we based our model on the shortest mean follow‐up observed (36 months in the GBA + DBS– cohort; Table 1), under which the model would be powered to detect a minimally detectable difference of 1.33 MoCA points between PD_GBA1+DBS+_ and PD_GBA1+DBS−_ at 3 years from baseline.

**TABLE 1:** Baseline Cohort Characteristics

Characteristics		GBA+DBS+	GBA+DBS−	GBA−DBS+	GBA−DBS−	*p*
		n = 28	n = 28	n = 40	n = 43	
Age at onset, mean (SD)		51 (10)	54 (10)	50 (9)	56 (8)	0.006
Age at baseline, mean (SD)		61 (9)	62 (10)	62 (8)	65 (7)	0.280
Disease duration at baseline, mean (SD)		10 (5)	8 (4)	12 (5)	9 (5)	0.003
Sex	F	9 (32%)	12 (43%)	12 (30%)	16 (37%)	0.727
	M	19 (68%)	16 (57%)	28 (70%)	27 (63%)	
Predominant motor symptoms	Left	17 (61%)	15 (54%)	23 (58%)	15 (35%)	0.081
	Right	11 (39%)	12 (43%)	16 (40%)	28 (65%)	
*GBA1* variant	Risk	13 (46%)	13 (46%)	WT	WT	≥ 0.999
	Mild	3 (11%)	4 (14%)			
	Severe	12 (42%)	11 (40%)			
Tau haplotype	H1/H1	15 (54%)	21 (75%)	24 (60%)	27 (63%)	0.624
	H1/H2	8 (29%)	7 (25%)	15 (38%)	13 (30%)	
	H2/H2	2 (7%)	–	1 (3%)	2 (5%)	
	H2/H3	1 (4%)	–	–	–	
ApoE haplotype	E2/E3	4 (14%)	3 (11%)	4 (10%)	7 (16%)	
	E2/E4	1 (4%)	1 (4%)	1 (3%)	‐	
	E3/E3	14 (50%)	19 (68%)	24 (60%)	24 (56%)	
	E3/E4	6 (21%)	5 (18%)	7 (18%)	9 (21%)	
	E3/E5	1 (4%)	‐	4 (10%)	‐	
PD subtype	Tremor	5 (18%)	4 (14%)	8 (20%)	3 (7%)	0.407
	Mixed	10 (36%)	16 (57%)	18 (45%)	24 (56%)	
	Akinetic	13 (46%)	8 (29%)	14 (35%)	16 (37%)	
MoCA, mean (SD)		27 (2)	26 (2)	28 (2)	27 (2)	0.016
MDS‐UPDRS III, mean (SD)		22 (10)	30 (11)	28 (12)	27 (12)	0.123
MDS‐UPDRS IV, mean (SD)		8 (7)	2 (3)	6 (3)	1 (1)	≤ 0.001
BDI II, mean (SD)		12 (10)	12 (5)	10 (6)	11 (5)	0.467
LEDD, mean (SD)		1,231 (553)	736 (445)	1,160 (547)	652 (413)	≤ 0.001
Follow‐up duration in months, mean (SD)		73 (50)	36 (21)	61 (26)	73 (36)	0.001
Mean follow‐up interval in months, mean (SD)		13 (14)	10 (9)	9 (9)	11 (11)	0.096

*P* values calculated with Kruskal‐Wallis test for nonparametric continuous variables and Fisher's exact test for categorial variables. Predominant motor symptoms: GBA+DBS− and GBA−DBS+ cohorts: in each cohort one patient with symmetrical motor symptoms.

BDI II = Beck Depression Inventory II; LEDD = Levodopa equivalent daily dosage [mg]; MDS‐UPDRS = Movement Disorders Society Unified Parkinson's Disease Rating Scale; MoCA = Montreal Cognitive Assessment; SD = standard deviation; WT = wild type.

As a secondary exploratory analysis, we analyzed the conversion to PD dementia. PD dementia was defined by a MoCA score < 21^26^, ^27^ together with evidence of cognitive deficits impairing independence in activities of daily living, as documented in structured routine clinical assessments, consistent with DSM‐5 criteria for a major neurocognitive disorder.^43^, ^44^ All cases were retrospectively screened for these criteria and PD dementia was determined accordingly. Time to PD dementia onset was assessed using Kaplan–Meier analysis. Group differences were tested with the Gehan–Breslow–Wilcoxon test and hazard ratios (HRs) were computed using the log rank test. Patients who did not develop PD dementia were censored at their last follow‐up visit.

Potential predictors of PD dementia were examined with Cox proportional hazards regression. For this, missing data were addressed using multiple imputation with the “auto” method in SPSS, generating 100 datasets under the missing‐at‐random assumption. Baseline variables without missing values were included as predictors but not imputed. Candidate predictors were selected based on prior literature.^45^, ^46^ Continuous variables were dichotomized by quartiles, with the upper or lower quartile serving as the reference category. Collinearity of variables was evaluated with Spearman's correlation; when *r* > 0.7, one variable of the correlated pair was excluded. Cox regression models were then fitted in each imputed dataset, and results were pooled according to Rubin's rules to derive HRs with 95% confidence intervals (CIs). Statistical significance was defined at an alpha‐level of ≤ 0.05.

## Results

### Baseline Characteristics

Table 1 summarizes baseline demographic, clinical, genetic, and CSF biomarker characteristics. Pairwise comparisons stratified by DBS status (Supplementary Table S2), *GBA1* carrier status (Supplementary Table S3), and *GBA1* variant severity (Supplementary Table S4). Disease duration did not differ when comparing PD_GBA1+_ cohorts (PD_GBA1+DBS+_ vs PD_GBA1+DBS−_, *p* = 0.247) but differed between PD_GBA1−_ cohorts (PD_GBA1−DBS+_ vs PD_GBA1−DBS−_, *p* ≤ 0.001; see Supplementary Table S2). Age at baseline was comparable among all cohorts. Both PD_GBA1_ cohorts were balanced concerning variant severity and all cohorts were comparable regarding the distribution of *MAPT* haplotypes and *ApoE4* alleles. A difference in baseline MoCA scores was observed comparing by *GBA1* status (PD_GBA1+DBS−_ vs PD_GBA1−DBS−_, *p* = 0.042; see Supplementary Table S3). Both cohorts undergoing STN‐DBS exhibited more pronounced motor fluctuations reflected by higher MDS‐UPDRS IV scores before surgery (PD_GBA1+DBS+_, *p* = 0.010 and PD_GBA1−DBS+_, *p* ≤ 0.001) and received higher LEDD (PD_GBA1+DBS+_, *p* ≤ 0.001 and PD_GBA1−DBS+_, *p* ≤ 0.001) compared with corresponding non‐DBS cohorts (see Supplementary Table S2). Follow‐up duration was longer in PD_GBA1+DBS+_ compared to PD_GBA1+DBS−_ (*p* = 0.002; see Supplementary Table S2). All cohorts showed comparable mean levels of CSF profiles of A*β*_1‐42_, h‐Tau, p181‐Tau, and NfL (Supplementary Fig S2). Propensity scores were calculated in order to equilibrate cohorts for potential confounders and weighted baseline characteristics are presented in Supplementary Table S5.

### Clinical Short‐Term Follow‐Up

The clinical status in the short‐term follow‐up after adapting STN‐DBS settings and reaching stable dopaminergic medications was assessed within a median time range of 12 to 14 months after STN‐DBS implantation (Table 2). The median change of MoCA scores did not differ significantly when compared among all cohorts (*p* = 0.268). Compared to pre‐operative baseline, the median LEDD decreased within the STN‐DBS implanted cohorts (PD_GBA1+DBS+_, *p* ≤ 0.001 and PD_GBA1−DBS+_, *p* ≤ 0.001) and did not differ in cohorts without STN‐DBS (PD_GBA1+DBS−_, *p* = 0.156 and PD_GBA1−DBS−_, *p* = 0.058).

**TABLE 2:** Changes in Scores During Short‐Term Follow‐Up

Characteristics	GBA+DBS+	GBA+DBS−	GBA−DBS+	GBA−DBS−	*p*
	n = 24	n = 27	n = 39	n = 39	
Months from baseline, median (95% CI)	14 (13 to 20)	12 (12 to 18)	13 (12 to 16)	13 (12 to 18)	0.171
Change in MoCA, median (95% CI)	−0.5 (−3.0 to 0.0)	+0.5 (−1.0 to 2.0)	−1.0 (−2.0 to 0.0)	−1.0 (−1.0 to 0.0)	0.268
Change in MDS‐UPDRS‐IV, median (95% CI)	−8.5 (−14.0 to 2.0)	0.0 (−8.0 to 13.0)	−10.0 (−14.0 to 0.0)	0.0 (−2.0 to 6.0)	0.077
Change in LEDD, median (95% CI)	−571 (−700 to −303)	+54 (0 to 120)	−460 (−550 to −249)	0 (0 to 153)	< 0.001

*P* values calculated with Kruskal‐Wallis test for nonparametric continuous variables.

95% CI = 95% confidence interval; LEDD = Levodopa equivalent daily dosage [mg]; MDS‐UPDRS = Movement Disorders Society Unified Parkinson's Disease Rating Scale; MoCA = Montréal Cognitive Assessment.

### Longitudinal Cognitive Decline Related to GBA1 Status and STN‐DBS Status

**FIGURE 1:**
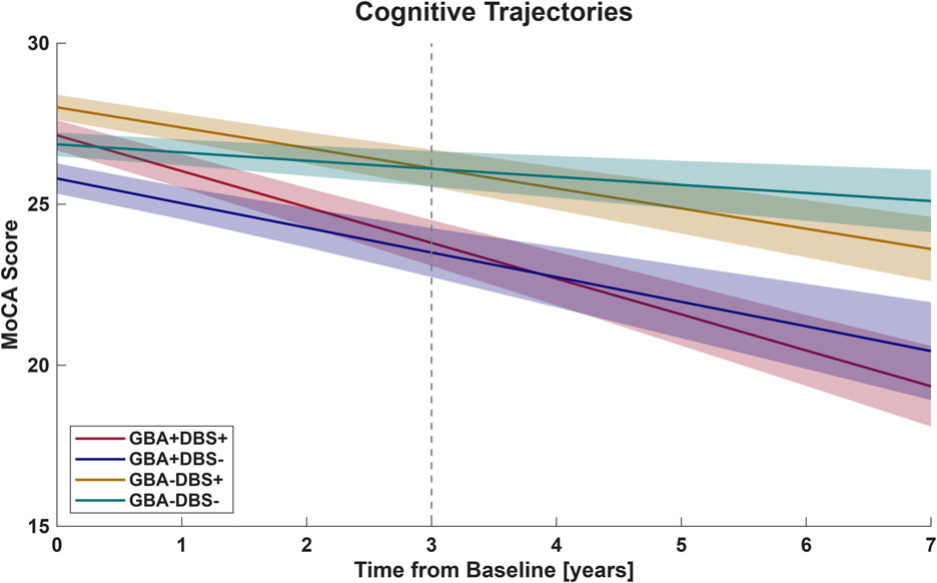
Linear fit of change in cognitive function assessed with the MoCA score over time upon *GBA1* and DBS status. PD_GBA1+DBS+_ declined by −1.1 points/year, PD_GBA1+DBS−_ by −0.9 points/year, PD_GBA1−DBS+_ by −0.6 points/year, and PD_GBA1−DBS−_ by −0.3 points/year. Time zero corresponds to the baseline screening visit ≤ 6 months before DBS implantation in the DBS cohorts with matched disease‐duration and patient age in the non‐DBS cohorts. Median follow‐up ranged from 42 to 90 months. Calculation was based on linear mixed modeling with random intercepts and slopes. The shaded areas represent the standard deviation of the mean projected groupwise slopes. The dashed line indicates the shortest mean follow‐up duration (PD_GBA1+DBS−_ = 36 months) used as reference for the power analysis. Baseline cognitive status represented as intercepts did not differ between PD_GBA1+DBS+_ versus PD_GBA1+DBS−_ (*p* = 0.054 and adjusted *p* [*p*_adj_] = 0.860) but differed significantly between PD_GBA1−DBS+_ versus PD_GBA1−DBS−_ (*p* = 0.026 and *p*_adj_ = 0.161) resolving after propensity score adjustment. DBS = deep brain stimulation; MoCA = Montreal Cognitive Assessment. [Color figure can be viewed at www.annalsofneurology.org]

Projected mean cognitive slopes for all cohorts are shown in Figure 1. Individual cognitive trajectories showed variability as demonstrated in Supplementary Figure S3. Patients with PD_GBA1+DBS+_ did not differ significantly from patients with PD_GBA1+DBS−_ (Table 3). The estimated slope difference was −0.24 MoCA points/year (95% CI = –1.11 to 0.70), corresponding to a projected −0.72 point difference at 3‐year follow‐up (*p* = 0.583). In contrast, patients with PD_GBA1–DBS+_ declined −0.39 MoCA points/year more than patients with PD_GBA1–DBS−_ (95% CI = −0.65 to −0.12, *p* = 0.005; Supplementary Fig S4).

**TABLE 3:** Pairwise Comparisons of Cognitive Development Assessed With the MoCA Among Cohorts Upon *GBA1* and DBS Status

	GBA+DBS−		GBA−DBS+		GBA−DBS−	
	Unadjusted [95% CI]	Adjusted [95% CI]	Unadjusted [95% CI]	Adjusted [95% CI]	Unadjusted [95% CI]	Adjusted [95% CI]
GBA+DBS+	−0.24	−0.26	−0.47	−0.43	−0.85	−0.93
	[−1.09 to 0.61]	[−1.21 to 0.7]	[−0.87 to −0.08]	[−1.0 to 0.2]	[−1.27 to −0.44]	[−1.43 to −0.43]
	*p* = 0.583	*p* = 0.593	*p* = 0.019*	*p* = 0.150	*p* < 0.001*	*p* < 0.001*
GBA+DBS−			−0.11	−0.06	−0.55	−0.56
			[−0.60, 0.38]	[−0.66 to 0.54]	[−0.99, −0.12]	[−1.02 to −0.11]
			*p* = 0.649	*p* = 0.850	*p* = 0.014*	*p* = 0.015*
GBA−DBS+					−0.39	−0.52
					[−0.65, −0.12]	[−0.82 to −0.21]
					*p* = 0.005*	*p* = 0.001*

Pairwise comparisons of the cohorts in rows in relation to the cohorts in columns are given as estimated additional loss of MoCA points/year [95% confidence interval], *P* value. *p < 0.05. The linear mixed model was adjusted for the logit of propensity scores integrating age at onset, age at baseline, baseline MoCA, baseline MDS‐UPDRS III, and baseline LEDD.

95% CI = 95% confidence interval; DBS = deep brain stimulation; LEDD = Levodopa equivalent daily dosage [mg]; MDS‐UPDRS = Movement Disorders Society Unified Parkinson's Disease Rating Scale; MoCA = Montreal Cognitive Assessment.

Comparisons by *GBA1* status revealed that carriers experienced stronger cognitive decline, regardless of STN‐DBS. PD_GBA1+DBS−_ declined −0.55 MoCA points/year more than PD_GBA1–DBS−_ (95% CI = −0.99 to −0.12, *p* = 0.014), and PD_GBA1+DBS+_ declined −0.47 MoCA points/year more than PD_GBA1–DBS+_ (95% CI = −0.87 to −0.08, *p* = 0.019; see Supplementary Fig S4). After propensity score adjustment, the difference in cognitive decline between PD_GBA1+DBS+_ and PD_GBA1−DBS+_ was no longer statistically significant (*p* = 0.150).

### Longitudinal Cognitive Decline Related to *GBA1* Variant Severity and STN‐DBS

**FIGURE 2:**
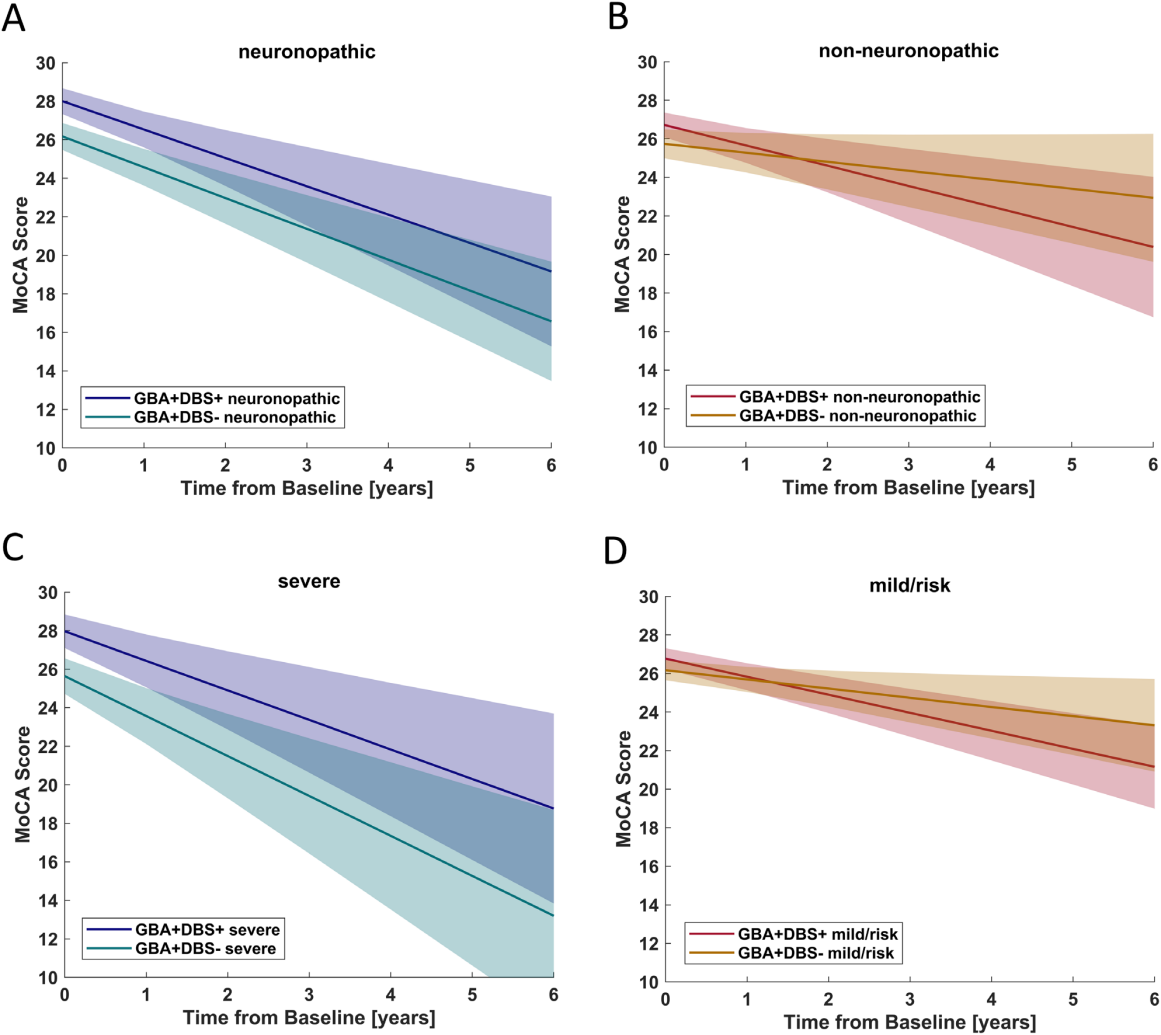
Pairwise comparisons of longitudinal cognitive trajectories assessed with the MoCA among cohorts stratified by *GBA1* variant severity. Comparison of patients with versus without STN‐DBS carrying (A) *GBA1* variants classified as neuronopathic (mild/severe), (B) *GBA1* variants classified as non‐neuronopathic (risk), (C) *GBA1* variants classified as severe, (D) pooled *GBA1* variants classified as mild/risk. Calculation was based on linear mixed modelling with random intercepts and slopes. The shaded areas represent the standard deviation of the mean projected groupwise slopes. Baseline cognitive status represented as intercepts did not differ significantly between groups. MoCA = Montreal Cognitive Assessment; STN‐DBS = subthalamic deep brain stimulation. [Color figure can be viewed at www.annalsofneurology.org]

Cohorts were stratified upon *GBA1* variant severity for exploratory intent, and longitudinal cognitive development was assessed in PD_GBA1+DBS+_ (n_severe_ = 12/28, n_mild_ = 3/28, and n_risk_ = 13/28) and PD_GBA1+DBS−_ (n_severe_ = 11/28, n_mild_ = 4/28, and n_risk_ = 13/28) subcohorts (Supplementary Fig S5). There was no difference in cognitive decline comparing patients with PD_GBA1+DBS+_ and patients with PD_GBA1+DBS−_ carrying *GBA1* variants classified as neuronopathic (relative cognitive decline = 0.28 MoCA points/year, 95% CI = −1.62 to 2.18, *p* = 0.773; Fig 2A) or non‐neuronopathic (relative cognitive decline = −0.54 MoCA points/year, 95% CI = −1.76 to 0.68, *p* = 0.383; Fig 2B). Among carriers of severe *GBA1* variants, there was no difference in cognitive decline between PD_GBA1+DBS+_ and PD_GBA1+DBS−_ (relative cognitive decline = −0.34 MoCA points/year, 95% CI = −2.67 to 3.35, *p* = 0.731; Fig 2C). Among carriers of pooled mild/risk variants there was no difference between PD_GBA1+DBS+_ and PD_GBA1+DBS−_ (relative cognitive decline = −0.72 MoCA points/year, 95% CI = −2.07 to 0.64, *p* = 0.267; Fig 2D).

### Onset of PD Dementia

**FIGURE 3:**
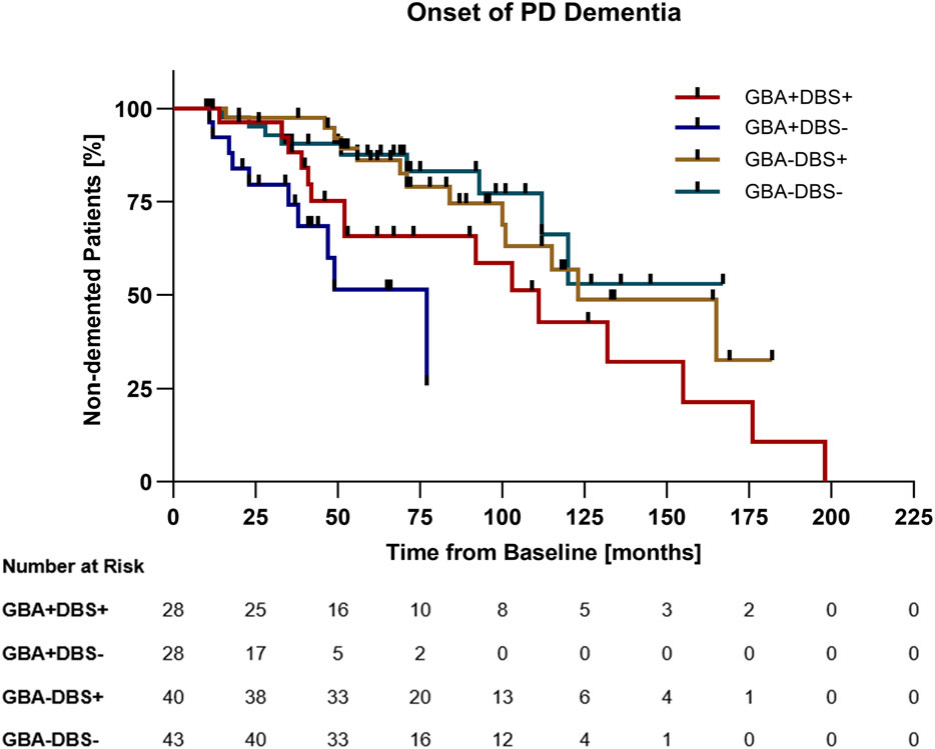
Kaplan–Meier analysis comparing dementia onset among cohorts. Patients with PD_GBA1+DBS+_ had a median time to PD dementia onset of 111 months (median follow‐up time 90 months). Patients with PD_GBA1+DBS−_ had a median time to PD dementia onset of 77 months (median follow‐up time = 42 months). PD_GBA1−DBS+_ had a median time to PD dementia onset of 123 months (median follow‐up time = 95 months). Less than 50% of PD_GBA1−DBS−_ developed PD dementia (median follow‐up time = 71 months). The baseline was defined as the screening visit ≤ 6 months before STN‐DBS implantation in the DBS+ cohorts with matched disease‐duration and patient age in the non‐DBS cohorts. Cases censored due to loss‐of follow up are listed as lines on the curves. PD = Parkinson's disease; STN‐DBS = subthalamic deep brain stimulation. [Color figure can be viewed at www.annalsofneurology.org]

In a secondary exploratory analysis, we studied the conversion to PD dementia (“event”) across cohorts (Fig 3). Among the 139 patients, a total of 47 events were observed (PD_GBA1+DBS+_: 15/28; PD_GBA1+DBS−_: 10/28; PD_GBA1−DBS+_: 13/40; and PD_GBA1−DBS−_: 9/43), whereas the remaining 92 patients were censored at the time of their last follow‐up visit. The HR for developing PD dementia comparing PD_GBA1+DBS+_ versus PD_GBA1+DBS−_ did not differ (HR = 0.55, 95% CI = 0.23 to 1.34, *p* = 0.119). Similarly, PD_GBA1–DBS+_ did not differ from PD_GBA1−DBS−_ (HR = 1.22, 95% CI = 0.53 to 2.83, *p* = 0.897). Among patients with STN‐DBS, PD_GBA1+DBS+_ did not differ from PD_GBA1−DBS+_ (HR = 1.75, 95% CI = 0.82 to 3.72, *p* = 0.067). In contrast, among patients without STN‐DBS, the PD_GBA1+DBS−_ cohort showed an increased risk of PD dementia compared with PD_GBA1−DBS−_ (HR = 3.85, 95% CI = 1.31 to 11.30, *p* = 0.004), as expected. Overall, *GBA1* variant carriers exhibited an earlier onset of dementia compared with non‐carriers, whereas STN‐DBS did not alter the hazard for conversion to PD dementia.

### Determinants of PD Dementia

**FIGURE 4:**
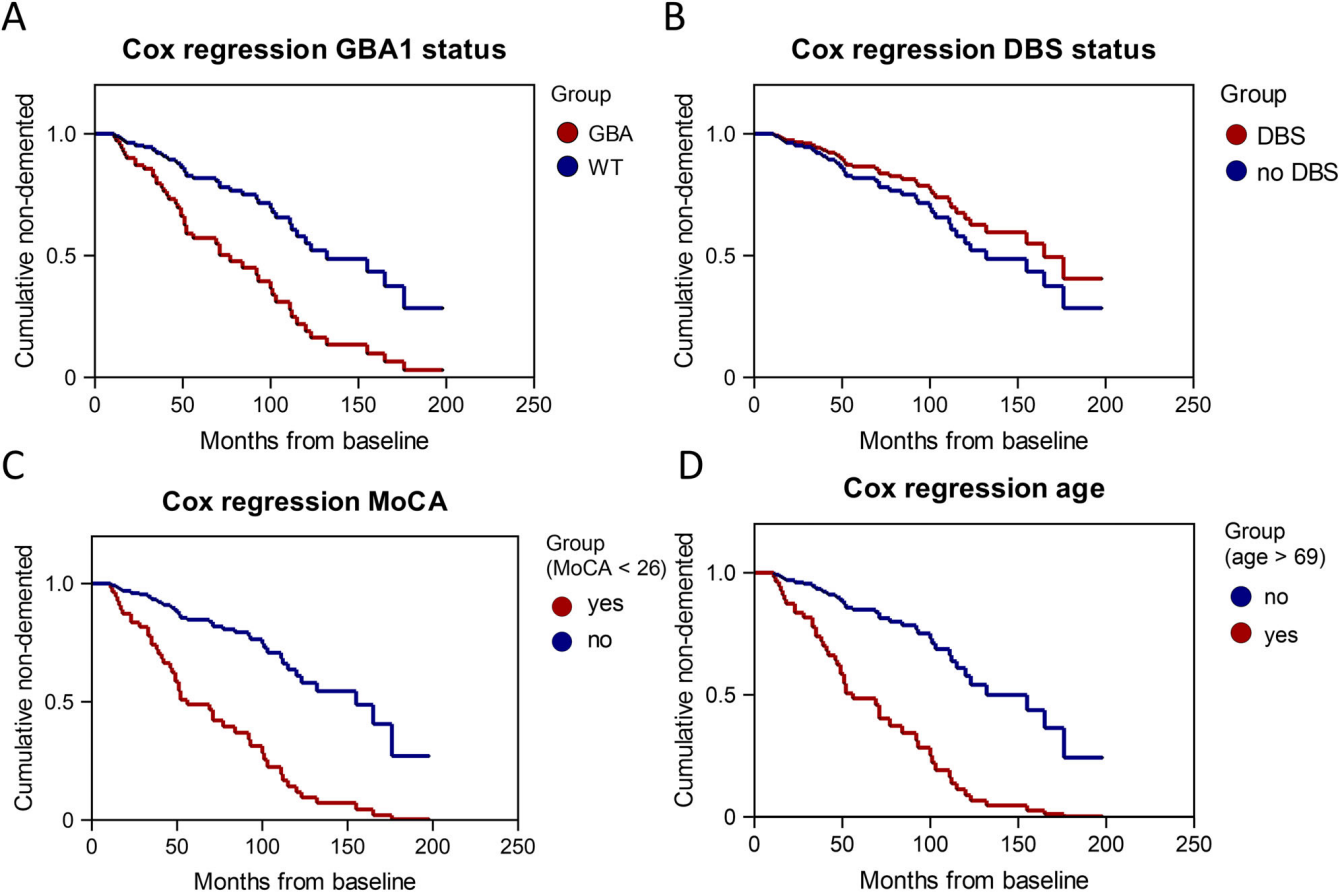
Cox regression analysis of clinical and biological determinants predicting PD dementia. The proportion of non‐demented patients over time (months) is displayed as survival curves grouped by (A) *GBA1* variant carriers versus WT, (B) DBS implanted versus non‐DBS implanted patients, (C) patients with an MoCA score below the first quartile (< 26 points) versus patients above the first quartile, (D) patient's age above the third quartile (> 69 years) versus below the third quartile. Cases censored due to loss‐of follow up are listed as circles on the curves. DBS = deep brain stimulation; MoCA = Montreal Cognitive Assessment; PD = Parkinson's disease; WT = wild type. [Color figure can be viewed at www.annalsofneurology.org]

Baseline determinants predicting later conversion to PD dementia were exploratorily analyzed using Cox regression (Table 4). Among all pooled patients, *GBA1* variants were associated with an increased hazard (HR = 3.04, 95% CI = 1.05 to 8.79, *p* = 0.041) predicting the onset of PD dementia (Fig 4A). However, the presence of STN‐DBS did not increase the hazard for PD dementia (HR = 0.71, 95% CI = 0.26 to 1.91, *p* = 0.491; Fig 4B). Moreover, patient age > 69 years (HR = 4.42, 95% CI = 1.79 to 10.89, *p* = 0.001) and a MoCA score < 26 (HR = 3.05, 95% CI = 1.29 to 7.21, *p* = 0.011) at baseline significantly increased the hazard for PD dementia (Fig 4C, D). Left‐sided motor symptoms^47^ were neither associated with more pronounced cognitive decline (Supplementary Fig S6) nor increased risk for PD dementia (see Table 4).

**TABLE 4:** Multivariate Cox Regression Analysis of Baseline Variables and Their Predictive Value for PD Dementia

	Hazard ratio	95% CI	*p*
*GBA1* variant carriers	3.04	1.05 to 8.79	0.041
STN‐DBS implantation	0.71	0.26 to 1.91	0.491
High disease duration (> 12 yr)	2.32	0.78 to 6.96	0.132
Higher age (> 69 yr)	4.42	1.79 to 10.89	0.001
Low MoCA (< 26 points)	3.05	1.29 to 7.21	0.011
High LEDD (> 1,315 mg/d)	0.74	0.23 to 2.33	0.605
High MDS‐UPDRS III (> 36 points)	1.24	0.42 to 3.67	0.698
High MDS‐UPDRS IV (> 5 points)	1.06	0.24 to 4.83	0.936
Predominantly left‐sided motor symptoms	0.85	0.61 to 1.20	0.354
Freezing of gait	0.69	0.22 to 2.21	0.532
Falls	0.93	0.29 to 3.00	0.901
Hallucinations	0.69	0.13 to 3.57	0.660
A*β*_1‐42_ pathology	0.81	0.22 to 3.08	0.761
NfL	1.41	0.36 to 5.52	0.621

Cutoff values for age, LEDD, MDS‐UPDRS III, and MDS‐UPDRS IV were determined as beyond the third quartile of the cohort for each respective variable. The cutoff value for the MoCA was determined as below the first quartile of the cohort. The cutoff value of A*β*1‐42 was < 600 pg/ml.

95% CI = 95% confidence interval; DBS = deep brain stimulation; LEDD = Levodopa equivalent daily dosage [mg]; MDS‐UPDRS = Movement Disorders Society Unified Parkinson's Disease Rating Scale; MoCA = Montréal Cognitive Assessment; NfL = neurofilament light chain; PD = Parkinson's disease.

### Cognitive Baseline Profile of Patients Developing PD Dementia

The cognitive profiles of PD_GBA1+_ and PD_GBA1−_ patients were analysed at baseline across MoCA subdomains, stratified by whether patients developed PD dementia during the subsequent disease course (Supplementary Fig S7). In PD_GBA1+_, patients subsequently developing PD dementia exhibited a significant baseline impairment in the visuospatial/executive domain compared with patients who maintained normal cognitive function throughout the disease course (*p* = 0.002). Specifically, a visuospatial/executive domain score of < 4 of 5 at baseline was associated with a significantly increased HR of 4.71 (95% CI = 1.25 to 17.80, *p* = 0.022) for developing PD dementia. Conversely, in PD_GBA1−_, baseline cognitive profiles did not differ between patients developing dementia and patients retaining regular cognitive function.

## Discussion

In this retrospective study, we analyzed longitudinal cognitive outcomes of matched cohorts of people with PD by assessing a rich set of clinical, genetic, and biological determinants. Specifically, patients were stratified into nested cohorts based on *GBA1* variant carrier status and implantation of STN‐DBS (PD_GBA1+DBS+_, PD_GBA1+DBS−_, PD_GBA1−DBS+_, and PD_GBA1−DBS−_), whereas pathogenic mutations in other PD‐related genes were not observed. Further, we included CSF profiles of A*β*_1‐42_, h‐Tau, p181‐Tau, and NfL providing not only clinical but also biological stratification, thereby controlling for potential confounders and identifying independent determinants of cognitive outcomes.

In primary analyses, we could not confirm an earlier report on steeper slopes of cognitive decline if PD_GBA1_ were treated with STN‐DBS.^12^ Additionally, *GBA1* variant carriers exhibited steeper cognitive decline irrespective of STN‐DBS status. This was corroborated by a secondary exploratory analysis of conversion to PD dementia, in which patients with PD_GBA1_ treated with STN‐DBS did not show an increased hazard for PD dementia. Further, we exploratively identified clinical baseline determinants of conversion to PD dementia, namely (i) patient age > 69 years and (ii) a MoCA score < 26. Specifically, in PD_GBA1+_ baseline cognitive status in patients subsequently developing PD dementia yielded a pattern of predominant visuospatial/executive impairment.

The controversy on whether cognitive decline in PD_GBA1_ accelerates in the presence of STN‐DBS has been nourished recently.^12^, ^21^ When comparing our findings to the original study,^12^ several key methodological differences must be considered. In our analysis, the difference in disease duration between the PD_GBA1+DBS+_ and PD_GBA1+DBS−_ cohorts was smaller, whereas, in the original study, this difference was larger, to the detriment of the PD_GBA1+DBS+_ group. Moreover, our STN‐DBS implanted cohorts were followed for a longer period, increasing the difference in follow‐up time between the DBS+ and non‐DBS cohorts. Although this may have led us to observe later disease progression related clinical changes in the DBS+ groups, it also helped to stabilize cognitive trajectories beyond early post‐OP effects.^17^ In addition, the relatively long observation time allowed us to study conversion to dementia as a secondary clinical end point supporting our findings. Notably, in cohorts with longer follow‐up, no increased hazard of PD dementia was observed, and the mean follow‐up interval was comparable between all cohorts, minimizing systematic bias of “data sampling.” Eventually, we also considered potential biological and genetic confounders improving biological comparability of the cohorts.

Compared to the original study, the PD_GBA1+_ cohorts were smaller in our analysis showing a nonsignificant cognitive slope difference of −0.72 MoCA points over 3 years between PD_GBA1+DBS+_ and PD_GBA1+DBS−_. Our model was sensitive enough to detect slope differences as small as 1.33 MoCA points over 3 years. Although we cannot exclude that the observed difference might have reached significance in a substantially larger cohort, the minimally clinically important difference of the MoCA has been estimated at ~2 points^48^, ^49^, ^50^ indicating that our model was adequately powered to detect clinically meaningful changes.

Concerning genetic cohort composition, our *GBA1* cohorts included a higher proportion of patients carrying severe *GBA1* variants (40%) compared to the 11% to 19% reported previously.^12^ In this context, exploratory stratification by *GBA1* variant severity did not indicate an effect of STN‐DBS on cognitive outcome in individuals carrying variants classified as severe or pooled neuronopathic thereby reflecting the most pronounced *GBA1*‐related disease biology.^51^, ^52^ However, these findings should only be regarded as preliminary given the small subgroup sizes limiting statistical power of this exploratory analysis. Here, further analyses of pooled multicenter datasets are essential to clarify the cognitive outcomes associated with specific *GBA1* variants in the context of STN‐DBS.

This study has limitations. When analyzing retrospective cognitive scores over a large time span from 2004 and 2023, we had to convert MMSE to MoCA scores in individual cases based on a validated algorithm,^33^ whereas the majority of our patients received serial MoCA assessments from the year 2014 on. We argue MMSE conversion may have impacted our findings to a minor degree. When predicting MoCA from MMSE scores ≥ 18 points as in this study, there may be small inaccuracy of up to 1 to 2 points within a 95% CI.^33^ This is comparable to the test–retest reliability when performing repeated MoCA assessments in a single patient^50^ and ranges within the threshold of the clinically meaningful difference of ~2 points.^48^, ^49^, ^50^ Regarding sample size, the linear mixed model was sufficiently powered to detect the clinically meaningful difference of 1.33 MoCA points 3 years from baseline. However, greater sample sizes and extended follow‐up, especially within the PD_GBA1+DBS−_ cohort, would have improved statistical sensitivity, given that cognitive slope estimates at longer follow‐up durations are based on progressively fewer participants. This is a limitation of the real‐world retrospective dataset which should be addressed in prospective future assessments of clinical data.

Furthermore, the analysis of conversion to PD dementia was likely underpowered due to a limited number of observed events to allow for confirmatory findings. Therefore, we treated these observations descriptively; that is, the observed direction of (numerically) less dementia conversion of the PD_GBA1+DBS+_ cohort compared with the PD_GBA1+DBS−_ cohort did not suggest a detrimental impact of STN‐DBS on cognitive outcomes in *GBA1* variant carriers. This hypothesis‐generating observation will have to be re‐evaluated in independent studies. Further, there are inherent limitations of the retrospective cohort design despite matching procedures. In clinical practice, patient selection for STN‐DBS is guided by well‐established eligibility criteria, primarily the presence of relevant motor fluctuations in the absence of substantial cognitive impairment.^25^, ^53^, ^54^ Consequently, patients who do not meet these criteria are generally not considered for STN‐DBS and therefore are not represented in this and the related studies.^6^, ^9^, ^12^ Cognitive selection prior to STN‐DBS may also explain why we found no statistically significant difference in cognitive decline between the PD_GBA1+DBS+_ and PD_GBA1−DBS+_ groups after propensity score adjustment – however, we found the expected difference between the DBS− cohorts when stratified by *GBA1* status consistent to previous findings.^1^, ^4^ Ultimately, this means that our conclusions do not apply to the entirety of *GBA1* variant carriers, but only to those that have widely preserved cognition once undergoing DBS eligibility screening.

Further, DBS+ cohorts showed increased MDS‐UPDRS IV scores and consequently a higher LEDD compared to DBS−, which may be considered as a clinical proxy of stronger presynaptic striatal denervation giving rise to fluctuations.^55^, ^56^, ^57^ Because of limited data availability in the DBS− cohorts, the baseline MDS‐UPDRS IV could not be included in the propensity score adjustment, representing a methodological limitation. Nevertheless, fluctuations and levodopa exposure have not been identified as predictors of cognitive decline in PD.^58^, ^59^ Instead, cognitive decline and in particular dementia are strongly linked to cholinergic network pathology^60^ including *GBA1* variant carriers who show a higher degree of cholinergic denervation in neurometabolic imaging at diagnosis compared to sporadic PD.^61^ Consistently, our exploratory sub‐analyses did not find an association between high MDS‐UPDRS IV scores or LEDD and an increased hazard of PD dementia. In this light, clinical stratification for STN‐DBS eligibility led to the inclusion of *GBA1* variant carriers exhibiting severe motor fluctuations while maintaining a widely preserved cognitive status. Indeed, the mean time to dementia onset in *GBA1* associated PD has been reported to be ~8.3 years compared to ~13.7 years in wildtype PD,^62^ thus our PD_GBA1+DBS+_ cohort (mean disease duration = 10 ± 5 years, MoCA = 27 ± 2) framed by DBS eligibility criteria may represent a sub‐phenotype among *GBA1* with relatively slow cognitive progression. This potential sub‐phenotype merits further investigation in future research, and the salience factors rendering a relevant proportion of *GBA1* variant carriers with more benign cognitive disease course are not well understood at present.

The fact that this cohort derives from a single center may limit the generalizability compared with multicenter approaches. However, the clinical, genetic, and biological stratification ensures comparability of cohorts at baseline and subsequent treatment of all patients at the same center under the same conditions stabilizes potential confounders related to the surgical procedure and postoperative management strategies. Regarding the representativeness of our cohort, our observations are consistent with previous findings showing that high patient age, impaired baseline cognitive function, and *GBA1* status were found as established risk factors for unfavorable cognitive outcome^1^, ^63^, ^64^, ^65^ in PD. However, we could not reproduce recent observations of more pronounced cognitive decline in patients with predominantly left‐sided motor symptoms.^47^ Another relevant limitation neither addressed in the original study^12^ nor in our analysis is that due to limited data availability, we could not account for anatomic DBS electrode placement, as electrode trajectories intersecting with the caudate nuclei can impact cognitive decline rates in the short‐term following STN‐DBS implantation.^66^, ^67^ In this setting, robust long‐term outcome parameters like PD dementia are of particular importance as they allow for an assessment of overall disease progression beyond immediate surgery‐related cognitive microlesion effects.^17^

Taken together and in the context of the above‐mentioned constraints given the limited sample size, the restricted ability to stratify by *GBA1* variant severity, and the partial reliance on converted MMSE scores, our data suggest that cognitive outcome prediction after STN‐DBS should not rely solely on *GBA1* carrier status but rather on an integrated clinical, genetic, and biomarker profile. When counseling for STN‐DBS, *GBA1* status may be considered a factor indicating a potential risk scenario for subsequent disease evolution, rather than a strict contraindication. Possible determinants such as baseline MoCA < 26, visuospatial or executive deficits, and age > 69 years may help identify individuals at greater risk of cognitive decline and inform shared decision making. Ultimately, confirmation of these observations requires larger, multicenter cohorts with comprehensive genetic and biomarker characterization, long‐term follow‐up, and eventually randomized controlled studies to assess cognition in *GBA1* variant carriers receiving STN‐DBS.

## Author Contributions

M.A.L., K.B. and D.W. contributed to the conception and design of the study; M.A.L., K.B. and D.W. contributed to the acquisition and analysis of data; M.A.L., K.B., D.W., I.W., S.L., P.K., I.C., T.G., and A.G. contributed to drafting the text or preparing the figures.

## Potential Conflicts of Interest

M.A.L., P.K., I.W., S.L., and K.B. declare nothing to report. A.G. and D.W. received research grants from Abbott, Boston Scientific, and Medtronic (manufacturers of DBS equipment, all not related to this work).

## Supporting information


**Supplementary Data S1.** Supplementary Figures and Tables.

## Data Availability

Anonymized clinical data that support the findings of this study are available from the corresponding authors upon reasonable request.
