# Supplementary material for: Clinical and Biological Determinants of Longitudinal Cognitive Function in Patients With GBA1 Variants and Subthalamic Deep Brain Stimulation
Source: Ann Neurol. 2026 Jan 16;99(4):976–88. doi: 10.1002/ana.78139 (PMC13011785; doi:10.1002/ana.78139)
Supplement: Supplementary file 1 — Supplementary Data S1. Supplementary Figures and Tables. [file ANA-99-976-s001.docx]

Clinical and biological determinants of longitudinal cognitive function in patients with *GBA1* variants and subthalamic deep brain stimulation

Supplemental Tables and Figures

Moritz A. Loeffler^1,2^, Philipp Klocke^1,2^, Isabel Wurster^1,2^, Stefanie Lerche^1,2^, Idil Cebi^1,2^, Thomas Gasser^1,2^, Alireza Gharabaghi^3^, Kathrin Brockmann^1,2^* and Daniel Weiss^1,2^*

*contributed equally

^1^ Centre for Neurology, Department of Neurodegenerative Diseases, University of Tübingen, Tübingen, Germany

^2^ Hertie-Institute for Clinical Brain Research, University of Tübingen, Tübingen, Germany

^3^ Institute for Neuromodulation and Neurotechnology, University of Tübingen, Tübingen, Germany

**Table of Contents:**

[Supplemental Table 1: Composition of *GBA1* cohorts regarding mutation profiles. 3](#_Toc209560632)

[Supplemental Figure 1: Assembly of the PDGBA+DBS+ cohort 4](#_Toc209560633)

[Supplemental Figure 2: Neurodegenerative markers in cerebrospinal fluid (CSF) in analysed cohorts 5](#_Toc209560634)

[Supplemental Table 2: Baseline cohort characteristics. Pairwise comparisons upon DBS+ vs. DBS- status. 6](#_Toc209560635)

[Supplemental Table 3: Baseline cohort characteristics. Pairwise comparisons upon *GBA1+* vs. *GBA1-* status. 7](#_Toc209560636)

[Supplemental Table 4: Baseline cohort characteristics. Pairwise comparisons upon *GBA1 severe* vs. *GBA1 mild/risk* status. 9](#_Toc209560637)

[Supplemental Table 5: Propensity score weighted baseline cohort characteristics. 10](#_Toc209560638)

[Supplemental Figure 3: Individual cognitive trajectories calculated in the Linear mixed model among groups 11](#_Toc209560639)

[Supplemental Figure 4: Pairwise comparisons of cognitive decline among cohorts upon GBA1 and DBS status 12](#_Toc209560640)

[Supplemental Figure 5: Explorative modelling of longitudinal cognitive outcome upon mutation severity 13](#_Toc209560641)

[Supplemental Figure 6: Motor symptom asymmetry and cognitive outcomes. 13](#_Toc209560642)

[Supplemental Figure 7: Baseline cognitive profile assessed with the Montreal Cognitive Assessment (MoCA) in GBA1 variant carriers and wildtype PD patients. 14](#_Toc209560643)

Supplemental Table 1: Composition of *GBA1* cohorts regarding mutation profiles.

| **Group** | **Severity** | **Mutation name** | **n** |
| --- | --- | --- | --- |
| **GBA+DBS+** | mild | p.D179H (D140H)-mild, p.E365K (E326K)-risk | 1 |
|  |  | p.N409S (N370S) | 2 |
|  | risk | p.E365K (E326K) | 9 |
|  |  | p.E365K (E326K), c.*92G>A, c.*102T>C | 1 |
|  |  | p.T408M (T369M) | 2 |
|  |  | E10 dup het, c.*92G>A | 1 |
|  | severe | p.D448H (D409H) | 1 |
|  |  | p.L483P (L444P) | 5 |
|  |  | p.L483P (L444P), p.A495P (A456P), p.V499= (V460V) | 1 |
|  |  | p.L483P (L444P), p.A495P (A456P), p.V499= (V460V), c.*92G>A, c.*102T>C | 1 |
|  |  | p.R398* (R359X) | 3 |
|  |  | p.D419N (D380N) | 1 |
| **GBA+DBS-** | mild | p.L307= (L268L), p.S310G (S271G) | 1 |
|  |  | p.N409S (N370S) | 3 |
|  | risk | E10 dup, p.T408M (T369M) hom | 1 |
|  |  | p.E365K (E326K) | 8 |
|  |  | p.T408M (T369M) | 4 |
|  | severe | c.1265_1319del | 1 |
|  |  | c.762-18T>A, p.L483P (L444P), p.A495P (A456P), p.V499= (V460V), c.*92G>A, c.*102T>C | 2 |
|  |  | p.E365K (E326K), p.N431S (N392S), p.L483P (L444P) | 1 |
|  |  | p.L483P (L444P) | 2 |
|  |  | p.L483P (L444P) (RecNcil), p.L483P (L444P), p.A495P (A456P), p.V499= (V460V), c.*92G>A, c.*102T>C | 1 |
|  |  | p.L483P (L444P), p.A495P (A456P), p.V499= (V460V), c.*92G>A, c.*102T>C | 1 |
|  |  | p.L483P (L444P), p.T449M (T410M) | 1 |
|  |  | p.P45L (P6L), p.R398* (R359X) | 1 |
|  |  | p.S164N (S125N) | 1 |


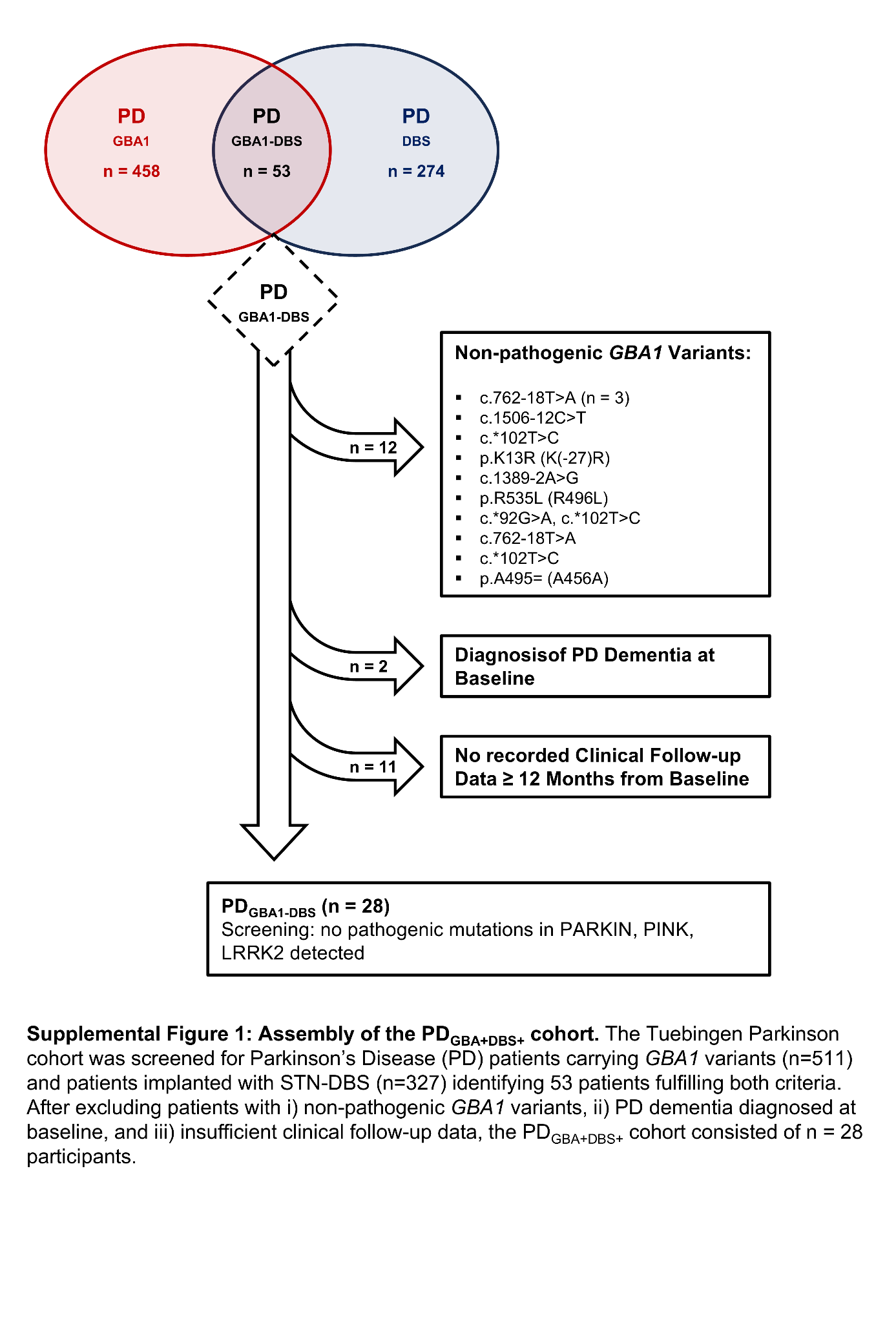


**Supplemental Figure 1: Assembly of the PDGBA+DBS+ cohort**. The Tuebingen Parkinson cohort was screened for Parkinson’s Disease (PD) patients carrying GBA1 variants (n=511) and patients implanted with STN-DBS (n=327) identifying 53 patients fulfilling both criteria. After excluding patients with i) non-pathogenic GBA1 variants, ii) PD dementia diagnosed at baseline, and iii) insufficient clinical follow-up data, the PDGBA+DBS+ cohort consisted of n = 28 participants.


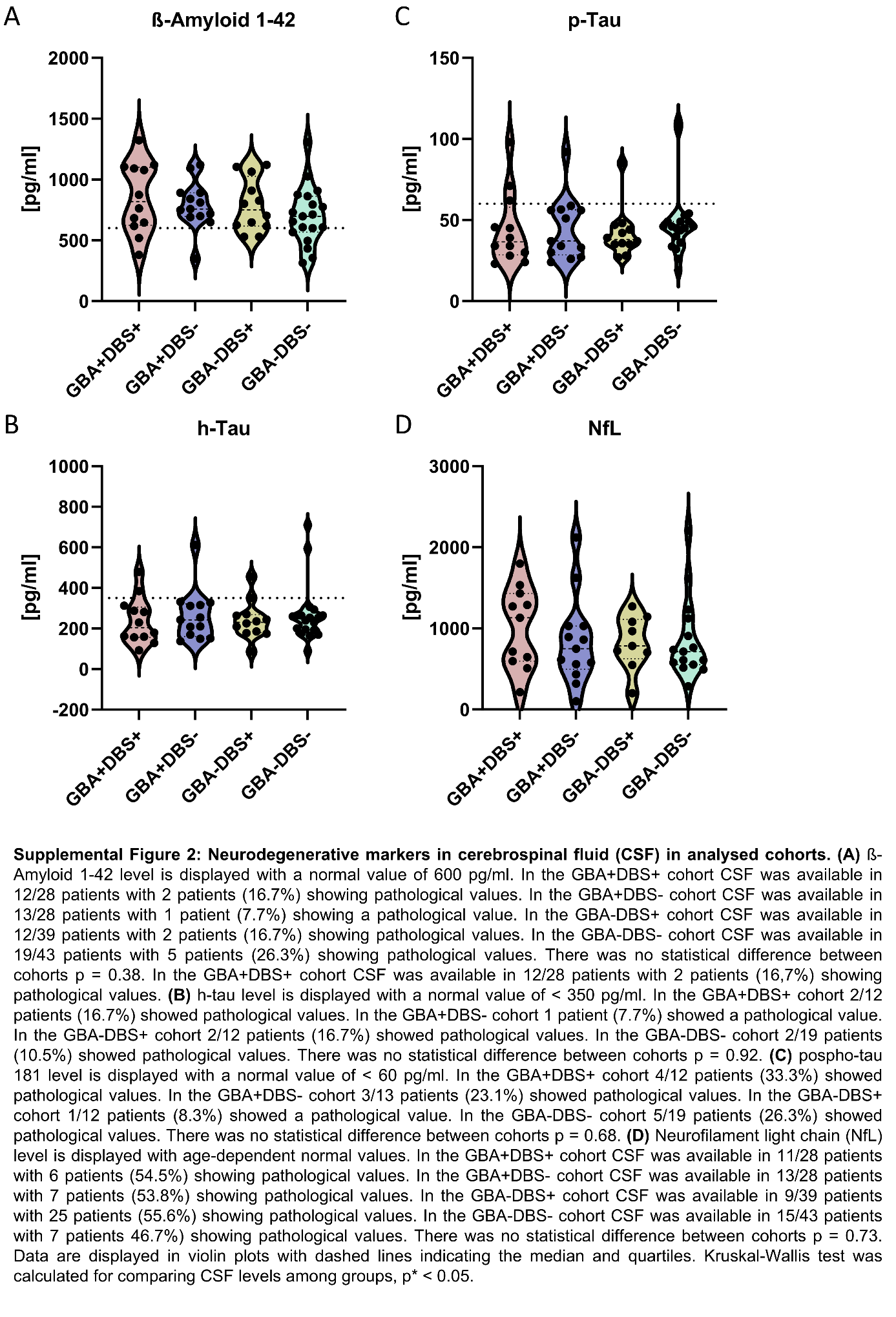


Supplemental Figure 2: Neurodegenerative markers in cerebrospinal fluid (CSF) in analysed cohorts. **(A)** ß-Amyloid 1-42 level is displayed with a normal value of 600 pg/ml or higher. In the GBA+DBS+ cohort CSF was available in 12/28 patients with 2 patients (16.7%) showing pathological values. In the GBA+DBS- cohort CSF was available in 13/28 patients with 1 patient (7.7%) showing a pathological value. In the GBA-DBS+ cohort CSF was available in 12/40 patients with 2 patients (16.7%) showing pathological values. In the GBA-DBS- cohort CSF was available in 19/43 patients with 5 patients (26.3%) showing pathological values. There was no statistical difference between cohorts p = 0.38. In the GBA+DBS+ cohort CSF was available in 12/28 patients with 2 patients (16,7%) showing pathological values. **(B)** h-tau level is displayed with a normal value of < 350 pg/ml. In the GBA+DBS+ cohort 2/12 patients (16.7%) showed pathological values. In the GBA+DBS- cohort 1 patient (7.7%) showed a pathological value. In the GBA-DBS+ cohort 2/12 patients (16.7%) showed pathological values. In the GBA-DBS- cohort 2/19 patients (10.5%) showed pathological values. There was no statistical difference between cohorts p = 0.92. **(C)** phospho-tau 181 level is displayed with a normal value of < 60 pg/ml. In the GBA+DBS+ cohort 4/12 patients (33.3%) showed pathological values. In the GBA+DBS- cohort 3/13 patients (23.1%) showed pathological values. In the GBA-DBS+ cohort 1/12 patients (8.3%) showed a pathological value. In the GBA-DBS- cohort 5/19 patients (26.3%) showed pathological values. There was no statistical difference between cohorts p = 0.68. **(D)** Neurofilament light chain (NfL) level is displayed with age-dependent normal values. In the GBA+DBS+ cohort CSF was available in 11/28 patients with 6 patients (54.5%) showing pathological values. In the GBA+DBS- cohort CSF was available in 13/28 patients with 7 patients (53.8%) showing pathological values. In the GBA-DBS+ cohort CSF was available in 9/39 patients with 25 patients (55.6%) showing pathological values. In the GBA-DBS- cohort CSF was available in 15/43 patients with 7 patients 46.7%) showing pathological values. There was no statistical difference between cohorts p = 0.73. Data are displayed in violin plots with dashed lines indicating the median and quartiles. Kruskal-Wallis test was calculated for comparing CSF levels among groups, p* < 0.05.

Supplemental Table 2: Baseline cohort characteristics. Pairwise comparisons upon DBS+ vs. DBS- status.

| **Characteristics** | | **GBA+DBS+** | **GBA+DBS-** | **P value** | **GBA-DBS+** | **GBA-DBS-** | **P value** |
| --- | --- | --- | --- | --- | --- | --- | --- |
|  |  | **n = 28** | **n = 28** |  | **n = 40** | **n = 43** |  |
| Age at onset, mean (SD) | | 51 (10) | 54 (10) | 0.294 | 50 (9) | 56 (8) | **< 0.001** |
| Age at baseline, mean (SD) | | 61 (9) | 62 (10) | 0.675 | 62 (8) | 65 (7) | 0.097 |
| Disease duration at baseline, mean (SD) | | 10 (5) | 8 (4) | 0.247 | 12 (5) | 9 (5) | **< 0.001** |
| Sex | f | 9 (32%) | 12 (43%) | 0.581 | 12 (30%) | 16 (37%) | 0.643 |
|  | m | 19 (68%) | 16 (57%) |  | 28 (70%) | 27 (63%) |  |
| Predominant motor symptoms | Left | 17 (61%) | 15 (54%) | 0.788 | 23 (58%) | 15 (35%) | **0.045** |
|  | right | 11 (39%) | 12 (43%) |  | 16 (40%) | 28 (65%) |  |
| GBA variant | risk | 13 (46%) | 13 (46%) | > 0.999 | WT | WT |  |
|  | mild | 3 (11%) | 4 (14%) |  |  |  |  |
|  | severe | 12 (42%) | 11 (40%) |  |  |  |  |
| Tau haplotype | H1/H1 | 15 (54%) | 21 (75%) | 0.223 | 24 (60%) | 27 (63%) | 0.727 |
|  | H1/H2 | 8 (29%) | 7 (25%) |  | 15 (38%) | 13 (30%) |  |
|  | H2/H2 | 2 (7%) | - |  | 1 (3%) | 2 (5%) |  |
|  | H2/H3 | 1 (4%) | - |  | - | - |  |
| ApoE haplotype | E2/E3 | 4 (14%) | 3 (11%) | 0.823 | 4 (10%) | 7 (16%) | 0.192 |
|  | E2/E4 | 1 (4%) | 1 (4%) |  | 1 (3%) | - |  |
|  | E3/E3 | 14 (50%) | 19 (68%) |  | 24 (60%) | 24 (56%) |  |
|  | E3/E4 | 6 (21%) | 5 (18%) |  | 7 (18%) | 9 (21%) |  |
|  | E3/E5 | 1 (4%) | - |  | 4 (10%) | - |  |
| PD subtype | tremor | 5 (18%) | 4 (14%) | 0.302 | 8 (20%) | 3 (7%) | 0.202 |
|  | mixed | 10 (36%) | 16 (57%) |  | 18 (45%) | 24 (56%) |  |
|  | akinetic | 13 (46%) | 8 (29%) |  | 14 (35%) | 16 (37%) |  |
| MoCA, mean (SD) | | 27 (2) | 26 (2) | 0.113 | 28 (2) | 27 (2) | 0.169 |
| MDS-UPDRS III, mean (SD) | | 22 (10) | 30 (11) | **0.026** | 28 (12) | 27 (12) | 0.690 |
| MDS-UPDRS IV, mean (SD) | | 8 (7) | 2 (3) | **0.010** | 6 (3) | 1 (1) | **< 0.001** |
| BDI-II, mean (SD) | | 12 (10) | 12 (5) | 0.120 | 10 (6) | 11 (5) | 0.778 |
| LEDD, mean (SD) | | 1231 (553) | 736 (445) | **< 0.001** | 1160 (547) | 652 (413) | **< 0.001** |
| Follow-up duration in months, mean (SD) | | 73 (50) | 36 (21) | **0.002** | 61 (26) | 73 (36) | 0.226 |
| Mean follow-up interval in months, mean (SD) | | 13 (14) | 10 (9) | **0.046** | 9 (9) | 11 (11) | 0.143 |
| SD: standard deviation  WT: wild type  MoCA: Montreal Cognitive Assessment  MDS-UPDRS: Movement Disorders Society Unified Parkinson‘s Disease Rating Scale  BDI-II: Beck Depression Inventory II  LEDD: Levodopa equivalent daily dosage [mg]  P values calculated with Mann-Whitney U Test for nonparametric continuous variables and Fisher’s exact test for categorial variables. Predominant motor symptoms: GBA+DBS- and GBA-DBS+ cohorts: in each cohort one patient with symmetrical motor symptoms. | | | | | | | |

Supplemental Table 3: Baseline cohort characteristics. Pairwise comparisons upon *GBA1+* vs. *GBA1-* status.

| **Characteristics** | | **GBA+DBS+** | **GBA-DBS+** | **P value** | **GBA+DBS-** | **GBA-DBS-** | **P value** |
| --- | --- | --- | --- | --- | --- | --- | --- |
|  |  | **n = 28** | **n = 40** |  | **n = 28** | **n = 43** |  |
| Age at onset, mean (SD) | | 51 (10) | 50 (9) | 0.685 | 54 (10) | 56 (8) | 0.206 |
| Age at baseline, mean (SD) | | 61 (9) | 62 (8) | 0.788 | 62 (10) | 65 (7) | 0.264 |
| Disease duration at baseline, mean (SD) | | 10 (5) | 12 (5) | 0.072 | 8 (4) | 9 (5) | 0.635 |
| Sex | f | 9 (32%) | 12 (30%) | > 0.999 | 12 (43%) | 16 (37%) | 0.804 |
|  | m | 19 (68%) | 28 (70%) |  | 16 (57%) | 27 (63%) |  |
| Predominant motor symptoms | Left | 17 (61%) | 23 (58%) | > 0.999 | 15 (54%) | 15 (35%) | 0.136 |
|  | right | 11 (39%) | 16 (40%) |  | 12 (43%) | 28 (65%) |  |
| GBA variant | risk | 13 (46%) | WT |  | 13 (46%) | WT |  |
|  | mild | 3 (11%) |  |  |  |  |  |
|  |  |  |  |  | 4 (14%) |  |  |
|  | severe | 12 (42%) |  |  | 11 (40%) |  |  |
| Tau haplotype | H1/H1 | 15 (54%) | 24 (60%) | 0.481 | 21 (75%) | 27 (63%) | 0.571 |
|  | H1/H2 | 8 (29%) | 15 (38%) |  | 7 (25%) | 13 (30%) |  |
|  | H2/H2 | 2 (7%) | 1 (3%) |  | - | 2 (5%) |  |
|  | H2/H3 | 1 (4%) | - |  | - | - |  |
| ApoE haplotype | E2/E3 | 4 (14%) | 4 (10%) | 0.848 | 3 (11%) | 7 (16%) | 0.578 |
|  | E2/E4 | 1 (4%) | 1 (3%) |  | 1 (4%) | - |  |
|  | E3/E3 | 14 (50%) | 24 (60%) |  | 19 (68%) | 24 (56%) |  |
|  | E3/E4 | 6 (21%) | 7 (18%) |  | 5 (18%) | 9 (21%) |  |
|  | E3/E5 | 1 (4%) | 4 (10%) |  | - | - |  |
| PD subtype | tremor | 5 (18%) | 8 (20%) | 0.655 | 4 (14%) | 3 (7%) | 0.552 |
|  | mixed | 10 (36%) | 18 (45%) |  | 16 (57%) | 24 (56%) |  |
|  | akinetic | 13 (46%) | 14 (35%) |  | 8 (29%) | 16 (37%) |  |
| MoCA, mean (SD) | | 27 (2) | 28 (2) | 0.139 | 26 (2) | 27 (2) | **0.042** |
| MDS-UPDRS III, mean (SD) | | 22 (10) | 28 (12) | **0.049** | 30 (11) | 27 (12) | **0.042** |
| MDS-UPDRS IV, mean (SD) | | 8 (7) | 6 (3) | 0.819 | 2 (3) | 1 (1) | 0.116 |
| BDI-II, mean (SD) | | 12 (10) | 10 (6) | 0.875 | 12 (5) | 11 (5) | 0.437 |
| LEDD, mean (SD) | | 1231 (553) | 1160 (547) | 0.958 | 736 (445) | 652 (413) | 0.419 |
| Follow-up duration in months, mean (SD) | | 73 (50) | 61 (26) | 0.106 | 36 (21) | 73 (36) | **< 0.001** |
| Mean follow-up interval in months, mean (SD) | | 13 (14) | 9 (9) | 0.622 | 10 (9) | 11 (11) | 0.342 |
| SD: standard deviation  WT: wild type  MoCA: Montreal Cognitive Assessment  MDS-UPDRS: Movement Disorders Society Unified Parkinson‘s Disease Rating Scale  BDI-II: Beck Depression Inventory II  LEDD: Levodopa equivalent daily dosage [mg]  P values calculated with Mann-Whitney U Test for nonparametric continuous variables and Fisher’s exact test for categorial variables. Predominant motor symptoms: GBA+DBS- and GBA-DBS+ cohorts: in each cohort one patient with symmetrical motor symptoms. | | | | | | | |

Supplemental Table 4: Baseline cohort characteristics. Pairwise comparisons upon *GBA1 severe* vs. *GBA1 mild/risk* status.

| **Characteristics** | | **GBA1 severe** | **GBA1 mild/risk** | **P value** |
| --- | --- | --- | --- | --- |
|  |  | **n = 23** | **n = 32** |  |
| Age at onset, mean (SD) | | 49 (10) | 55 (9) | **0.013** |
| Age at baseline, mean (SD) | | 59 (9) | 64 (9) | **0.047** |
| Disease duration at baseline, mean (SD) | | 9 (5) | 8 (4) | 0.345 |
| Gender | f | 9 | 12 | 0.526 |
|  | m | 14 | 21 |  |
| Predominant motor symptoms | Left | 17 | 15 | 0.054 |
|  | right | 6 | 17 |  |
| Tau haplotype | H1/H1 | 12 | 24 | 0.440 |
|  | H1/H2 | 8 | 7 |  |
|  | H2/H2 | 1 | 1 |  |
|  | H2/H3 | 1 | - |  |
| ApoE haplotype | E2/E3 | 4 | 3 | 0.694 |
|  | E2/E4 | - | 2 |  |
|  | E3/E3 | 13 | 20 |  |
|  | E3/E4 | 4 | 7 |  |
|  | E3/E5 | 1 |  |  |
| PD subtype | tremor | 3 | 6 | 0.567 |
|  | mixed | 12 | 14 |  |
|  | akinetic | 7 | 13 |  |
| MoCA, mean (SD) | | 26 (3) | 27 (2) | 0.853 |
| MDS-UPDRS III, mean (SD) | | 26 (11) | 27 (12) | 0.792 |
| MDS-UPDRS IV, mean (SD) | | 7 (7) | 3 (4) | 0.095 |
| BDI-II, mean (SD) | | 14 (8) | 10 (5) | **0.042** |
| LEDD, mean (SD) | | 933 (452) | 1015 (618) | 0.979 |
| SD: standard deviation  WT: wild type  MoCA: Montreal Cognitive Assessment  MDS-UPDRS: Movement Disorders Society Unified Parkinson‘s Disease Rating Scale  BDI-II: Beck Depression Inventory II  LEDD: Levodopa equivalent daily dosage [mg]  P values calculated with Mann-Whitney U Test for nonparametric continuous variables and Fisher’s exact test for categorial variables. Predominant motor symptoms: GBA+DBS- and GBA-DBS+ cohorts: in each cohort one patient with symmetrical motor symptoms. | | | | |

Supplemental Table 5: Propensity score weighted baseline cohort characteristics.

| **Characteristics** | | **GBA+DBS+** | **GBA+DBS-** | **GBA-DBS+** | **GBA-DBS-** |
| --- | --- | --- | --- | --- | --- |
| Age at onset, mean (SD) | | 55 (11) | 53 (8) | 53 (7) | 57 (6) |
| Age at baseline, mean (SD) | | 64 (9) | 63 (8) | 66 (8) | 67 (7) |
| Disease duration at baseline, mean (SD) | | 8 (6) | 9 (4) | 12 (6) | 13 (6) |
| Sex | f | 22 (41%) | 19 (37%)) | 21 (33%) | 24 (36%) |
|  | m | 31 (59%) | 32 (63%) | 43 (67%) | 43 (64%) |
| Predominant motor symptoms | left | 33 (63%) | 27 (53%) | 42 (66%) | 24 (37%) |
|  | right | 19 (37%) | 22 (44%) | 22 (34%) | 42 (63%) |
| MoCA, mean (SD) | | 26 (2) | 26 (2) | 27 (2) | 27 (2) |
| MDS-UPDRS III, mean (SD) | | 22 (9) | 29 (11) | 26 (12) | 30 (13) |
| MDS-UPDRS IV, mean (SD) | | 6 (7) | 2 (3) | 3 (3) | 2 (4) |
| BDI-II, mean (SD) | | 13 (11) | 12 (5) | 9 (5) | 13 (6) |
| LEDD, mean (SD) | | 1000 (393) | 949 (582) | 792 (450) | 835 (400) |
| SD: standard deviation  WT: wild type  MoCA: Montreal Cognitive Assessment  MDS-UPDRS: Movement Disorders Society Unified Parkinson‘s Disease Rating Scale  BDI-II: Beck Depression Inventory II  LEDD: Levodopa equivalent daily dosage [mg]  Propensity scores were derived from a binary logistic regression incorporating age at onset, age at baseline, baseline MoCA, baseline MDS-UPDRS III, and baseline LEDD as covariates, with STN-DBS (yes/no) as binary dependent variable. The dataset was weighted for these propensity scores. Nominal variables are displayed as a number (percentage rounded). Time variables are displayed in years, mean (standard deviation). | | | | | |


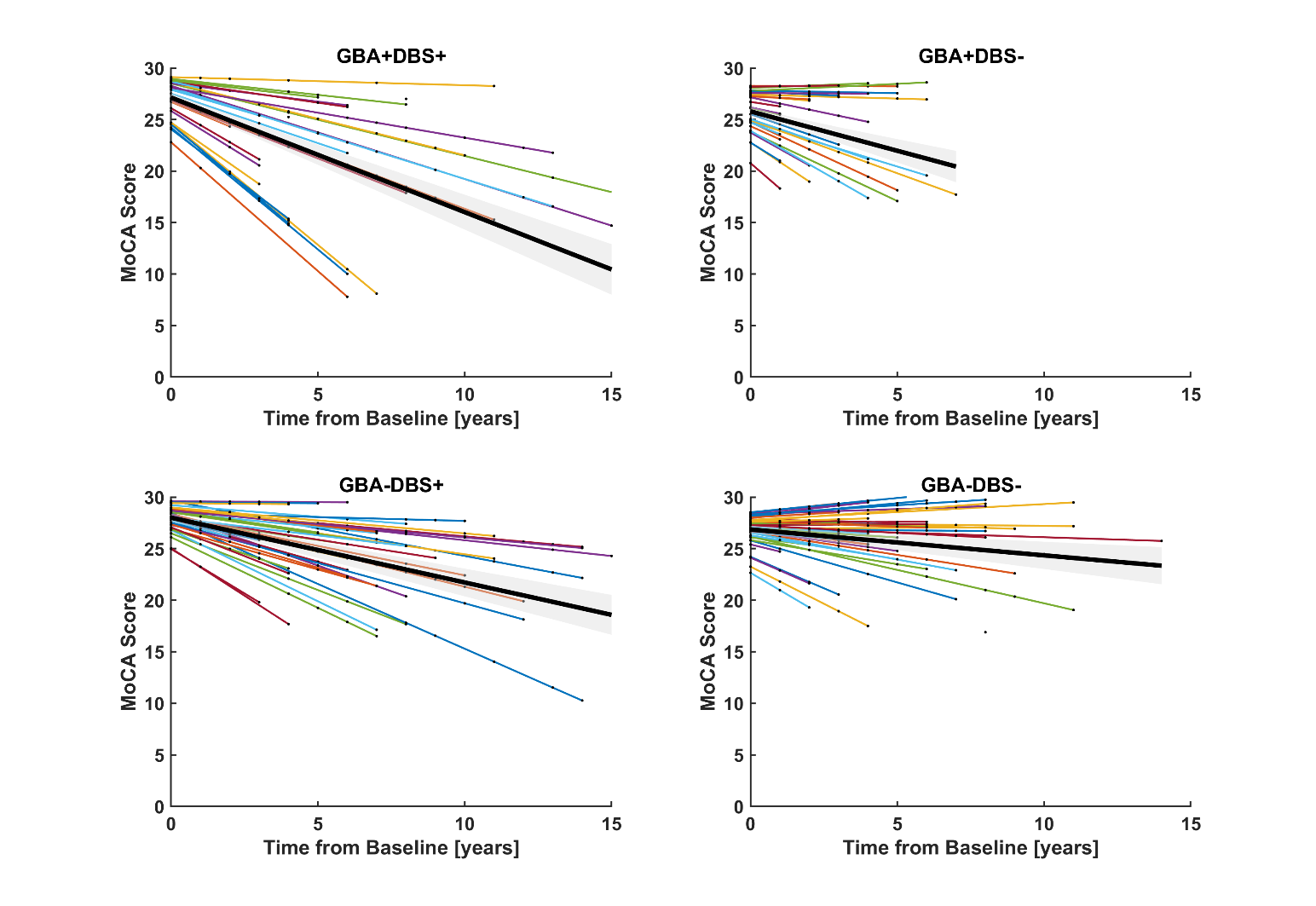


Supplemental Figure 3: Individual cognitive trajectories calculated in the Linear mixed model among groups. Time zero corresponds to baseline screening visit ≤ 6 months before deep brain stimulation (DBS) implantation in the DBS cohorts and matched disease-duration and patient age in the non-DBS cohorts. Median follow-up ranged from 42 – 90 months. Calculation was based on linear mixed-modelling with random intercept and slope. Coloured lines represent individual cognitive trajectories, and bold lines represent groupwise fixed trajectories calculated in the model, with the shaded area representing the standard deviation.


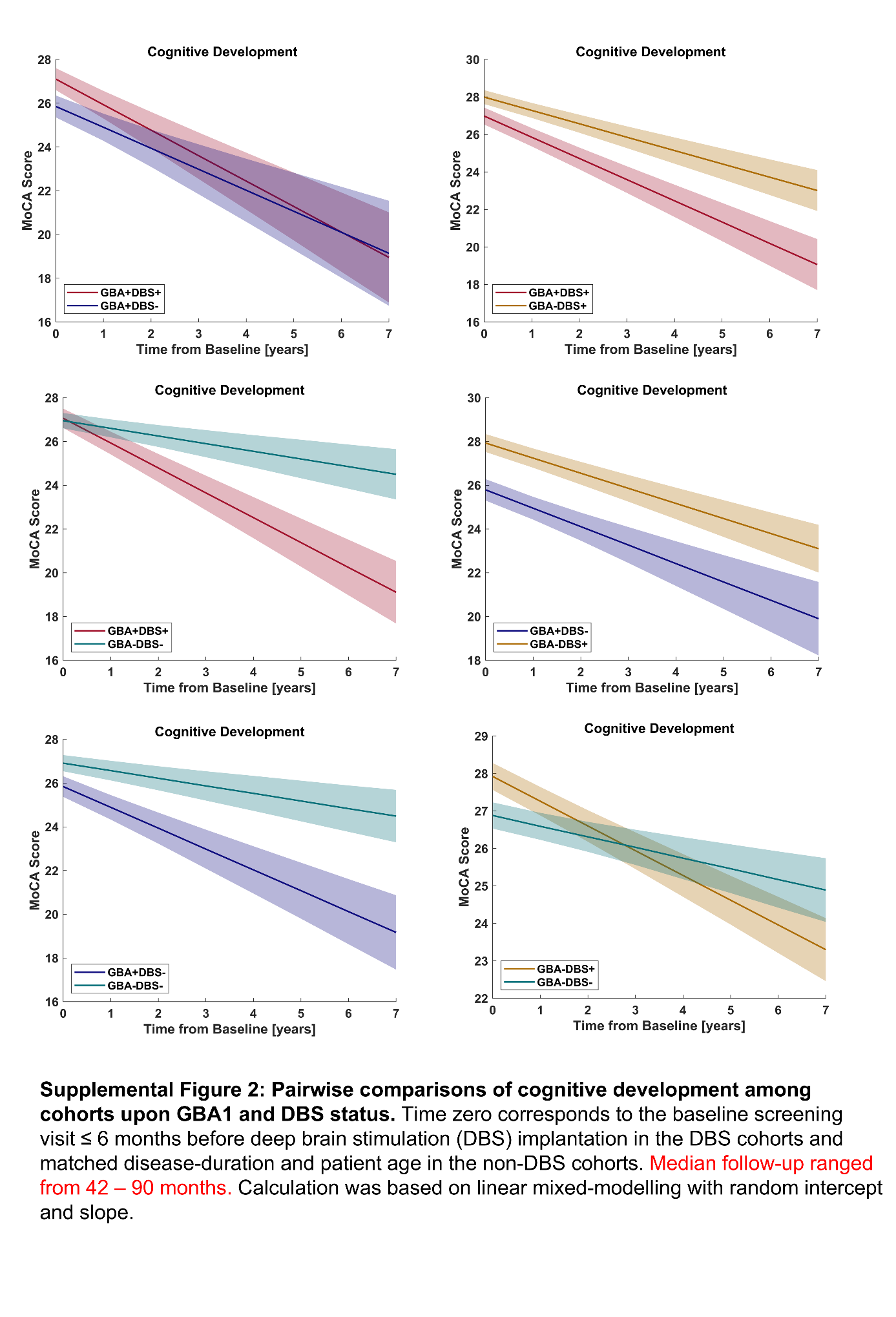


Supplemental Figure 4: Pairwise comparisons of cognitive decline among cohorts upon *GBA1* and DBS status. Time zero corresponds to baseline screening visit ≤ 6 months before deep brain stimulation (DBS) implantation in the DBS cohorts and matched disease-duration and patient age in the non-DBS cohorts. Median follow-up ranged from 42 – 90 months. Calculation was based on linear mixed-modelling with random intercept and slope. Baseline cognitive status represented as intercepts significantly differed comparing PD_GBA1+DBS-_ vs. PD_GBA1-DBS+_ (p = 0.005) and _PDGBA+DBS-_ vs. PD_GBA1-DBS-_ (p = 0.012).

**
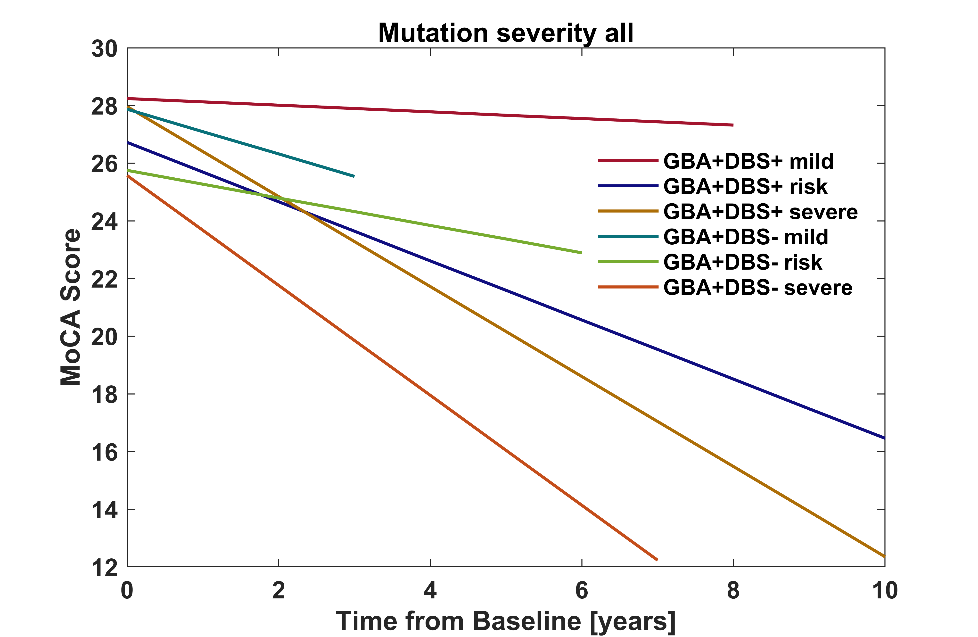
**

Supplemental Figure 5: Explorative modelling of longitudinal cognitive outcome upon mutation severity. GBA+DBS+ participants carrying mild mutations declined by 0.12 MoCA points/year. GBA+DBS+ participants carrying risk mutations declined by 1.0 MoCA points/year. GBA+DBS+ participants carrying severe mutations declined by 1.56 MoCA points/year. GBA+DBS- participants carrying mild mutations declined by 0.78 MoCA points/year. GBA+DBS- participants carrying risk mutations declined by 0.48 MoCA points/year. GBA+DBS- participants carrying severe mutations declined by 1.96 MoCA points/year.


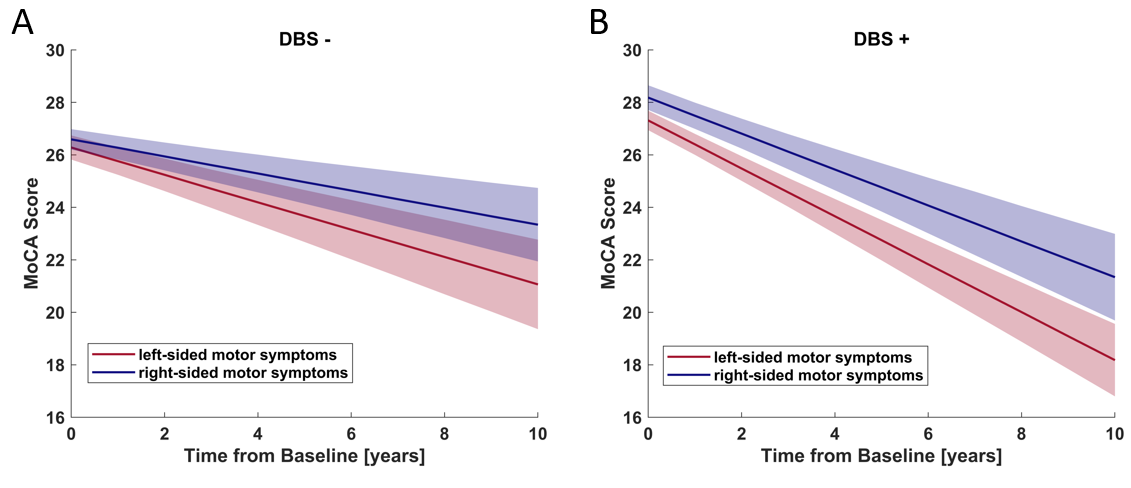


Supplemental Figure 6: Motor symptom asymmetry and cognitive outcomes. **(A)** In participants without subthalamic nucleus deep brain stimulation (DBS−), 30 individuals (42.3%) exhibited predominantly left-sided motor symptoms, while 40 (56.3%) exhibited predominantly right-sided symptoms. There was no statistically significant difference in the rate of cognitive decline between groups; those with left-sided symptoms declined by an additional −0.196 MoCA points per year (95% CI: −0.596; 0.204; *p* = 0.323). **(B)** In participants with STN-DBS (DBS+), 39 individuals (59.1%) had predominantly left-sided symptoms, and 26 (39.4%) had predominantly right-sided symptoms. Similarly, no significant difference in annual cognitive decline was observed; those with left-sided symptoms declined by an additional −0.229 MoCA points per year (95% CI: −0.632; 0.174; *p* = 0.264).


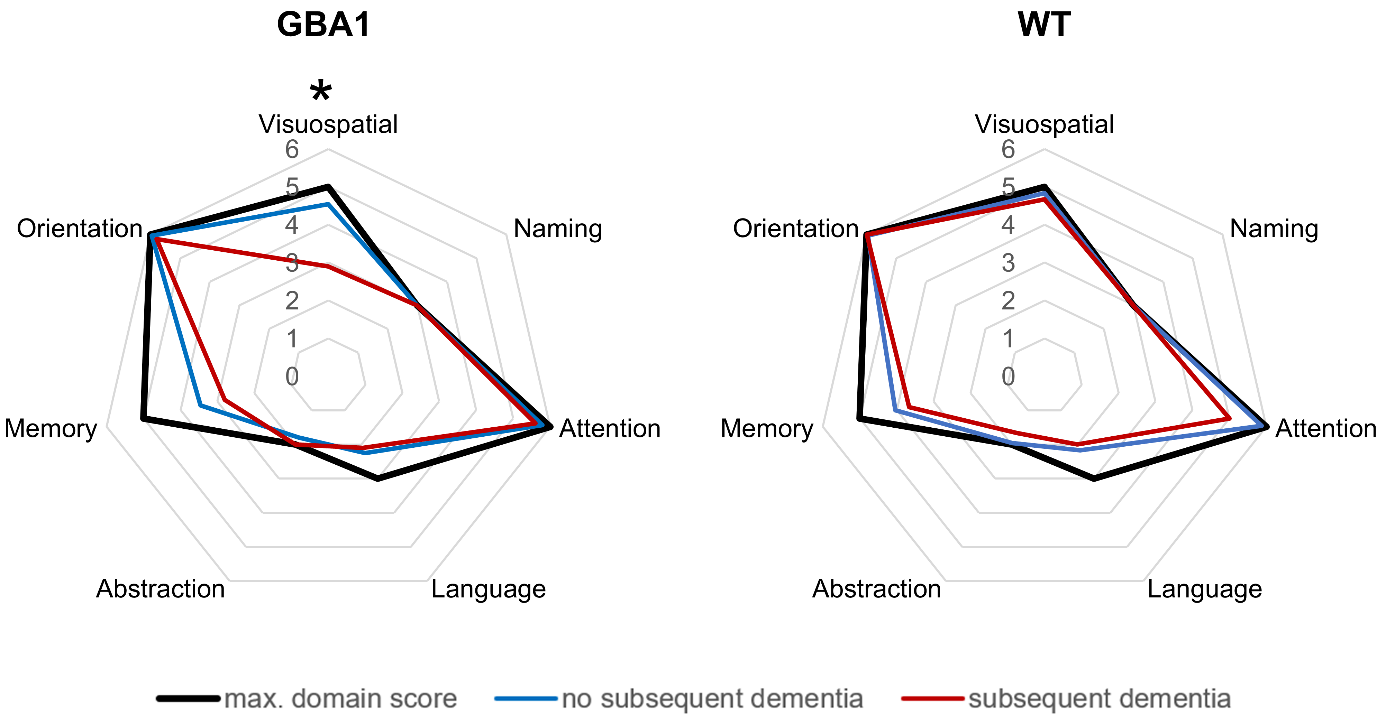


Supplemental Figure 7: Baseline cognitive profile assessed with the Montreal Cognitive Assessment (MoCA) in GBA1 variant carriers and wildtype PD patients. The baseline cognitive status was assessed using the MoCA score including domain scores (black line – maximum score achievable in each domain) in patients developing dementia (red line – subsequent dementia) and in patients without dementia (blue line – no subsequent dementia) in the subsequent disease course. P* < 0.05.
